# The Use of Plant Antimicrobial Compounds for Food Preservation

**DOI:** 10.1155/2015/246264

**Published:** 2015-10-11

**Authors:** Tana Hintz, Karl K. Matthews, Rong Di

**Affiliations:** ^1^Department of Food Science, Rutgers, The State University of New Jersey, New Brunswick, NJ 08901, USA; ^2^Department of Plant Biology, Rutgers, The State University of New Jersey, New Brunswick, NJ 08901, USA

## Abstract

Foodborne disease is a global issue with significant impact on human health. With the growing consumer demand for natural preservatives to replace chemical compounds, plant antimicrobial compounds must be thoroughly investigated for their potential to serve as biopreservatives. This review paper will focus on the plant-derived products as antimicrobial agents for use in food preservation and to control foodborne pathogens in foods. Structure, modes of action, stability, and resistance to these plant compounds will be discussed as well as their application in food industries and possible technologies by which they can be delivered. Benefits as well as challenges, such as the need for further research for implementation and governmental regulation, will be highlighted.

## 1. Introduction

The use of plants for healing dates to prehistory. As early as 60,000 years ago, the Neanderthals, in present-day Iraq, used plants including hollyhock for healing. These plants are still used globally [1]. Hippocrates wrote about several hundred medicinal plants in the late fifth century B.C. and the Bible mentions healing plants, such as frankincense and myrrh, which have antiseptic properties [1]. Plant oils and plant extracts have been utilized for thousands of years, serving many purposes, such as food preservatives and medical therapeutic agents [2]. The compounds that are found in some spices and produced by herbs act as self-defense mechanisms to protect the plant against infectious organisms [3]. They are also used by many cultures as flavoring agents and as natural preservatives in food. For example, in foods of India and in traditional Indian medicine, many spices, including garlic, black pepper, cumin, clove, ginger, and caraway, are used [4].

The majority of western plant pharmaceutical information was destroyed during the fall of ancient civilizations, but the Renaissance saw a revival in the use of medicinal plants in the western world [1]. In North America, indigenous cultures have used medicinal plants since prehistory and Americans of European origin began using botanicals in the 19th century to counter the toxic medical practices of that time, such as the use of mercury baths to treat syphilis [1].

Asian culture focuses on the use of herbs to treat diseases and illnesses. Throughout China's history, extensive research was conducted to learn the curative powers of plants. The* Imperial Grace Formulary* that was compiled in 985 C.E. contains 16,834 herbal entries. Indeed, Chinese medicine reflects traditions developed over 3,000 years and is a holistic approach that takes into account a condition in relation to the whole body in contrast to Western medicine that focuses on a specific cause and attempts to control it. Although Chinese and Western medicine are based on very different philosophies, Chinese herbal medicine has become well-known in the US and in Britain over the last few decades [5].

## 2. Approaches to Control Bacteria: Human Health and Food

Food safety is a global issue with significant implications for human health. The World Health Organization reports that, annually, unsafe food results in the illnesses of at least 2 billion people worldwide and can be deadly. Some countries have made great progress in controlling foodborne diseases, but the number of those affected by foodborne diseases is growing globally (WHO, 2004). In the United States, the Centers for Disease Control and Prevention (CDC) estimate that each year about 1 in 6 Americans becomes ill and thousands die of foodborne diseases (http://www.cdc.gov/foodborneburden/2011-foodborne-estimates.html).

Thermal processing is a common method of destroying vegetative microorganisms to ensure food safety, but this technique may cause undesirable nutritional and quality effects [3]. Preservatives are commonly used to reduce the risk of foodborne illnesses. Increasing regulatory restrictions and consumer negative response to chemical compounds and to the use of antibiotics in agriculture have contributed to the pressure for the development of alternative compounds for use as antimicrobial agents [6].

Antimicrobial agents have been predominantly isolated from bacteria and fungi and either produced through fermentation or produced chemically [1]. In the United States, one-quarter to one-half of pharmaceuticals are derived from plants, but very few are used as antimicrobials. Worldwide, spending on anti-infective agents has increased in recent years due to the limited effective lifespan of antibiotics as new resistant microbes emerge [1]. New sources, including plants, must be thoroughly investigated for identification of novel antimicrobial compounds. For example, it is known that some spices and herbs confer antimicrobial activity. Although there are conflicting reports in the literature about the absolute efficacy of various spices and herbs, Holley and Patel [7] state that the spices, cinnamon, mustard, vanillin, clove, and allspice, and some herbs, specifically oregano, rosemary, thyme, sage, and basil, all confer strong antimicrobial activity. They continue by stating that there are many others that show limited or moderate antimicrobial activity as well. However, Nychas [8] suggests that Gram-positive bacteria are generally more sensitive than Gram-negative bacteria to the antimicrobial compounds of spices.

Alternatives to traditional antimicrobial compounds include bacteriophages, antimicrobial phytochemicals, and antimicrobial peptides. There is less extensive research on the application of antimicrobial compounds from sources other than bacteria. This review paper will focus on the use of plant products as antimicrobial agents, specifically for use in food preservation and safety. Given the consumer demand for natural preservatives and the rapid rate of plant species extinction, it is imperative that more research is focused on the application of plant antimicrobials to food safety.

## 3. Plant Antimicrobial Peptides (pAMPs)

First discovered in 1942, antimicrobial peptides are produced by bacteria, animals, and plants to serve as natural defense compounds against pathogens. Although generally accepted to be small molecules, there is debate amongst researchers about their exact size. According to Joerger [6], they have a molecular mass between 1 and 5 kDa, but, according to Choon Koo et al. [9] and Garcia-Olmedo et al. [10], they range in size between 2 and 9 kDa. Furthermore, they are predominantly positively charged and are amphiphilic [11]. Although there are many sources of AMPs, this section will focus on plant-derived antimicrobial peptides and their modes of action.

Research clearly demonstrates that antimicrobial peptides are a key part of a plant's defense against pathogens, serving roles both in the defense response upon infection and as part of the preexisting defense barrier. Research has demonstrated that a peptide serves a defense role based on whether peptide-sensitive mutants of pathogens show decreased virulence in plant tissues containing the respective peptide and/or whether overexpression of the peptide results in enhanced tolerance of the plant to the pathogen [10]. Some peptides show specificity towards Gram-negative or Gram-positive bacteria, but most are able to inhibit the activity of both [12]. Plant AMPs are predominantly cysteine-rich compounds that have been isolated from different plant species and from different tissues. A recent review has listed more than twenty different pAMPs [13]. Although there is debate amongst researchers about the number of families, they can be divided into distinct protein families based on structure and amino acid sequence characteristics [9]. These plant antimicrobial peptide families are shown below.

### 3.1. Thionins

The first discovered pAMPs, thionins, are toxic to yeast, fungi, and Gram-positive and Gram-negative bacteria [14]. The example of wheat purothionin-*α*1 [15] is shown in Table 1 with 45 amino acids. They are able to induce leakage of intracellular material in bacteria and yeast. Regarding mode of action, it has been shown that they cause cell permeability to isoaminobutyric acid and affect electrical currents in artificial membranes. Purified genetic variants of thionins exhibited differences in activity and some differences in specificity [10]. López-Solanilla et al. [16] used an* in vitro* method to show that thionins purified from wheat flour have a strong inhibitory effect on multiple strains of* Listeria monocytogenes* and on a strain of* Listeria ivanovii*, which is another pathogenic species of the genus* Listeria*. The MICs (minimum inhibitory concentrations) are listed in Table 2. The authors also studied the effect of temperature on listerial susceptibility to AMPs and determined that a shift from environmental temperature (20°C) to mammalian host temperature (37°C) made* L. monocytogenes* more sensitive to thionin, but the opposite was shown for* L. ivanovii*.

### 3.2. Plant Defensins

Structurally related to insect and mammalian defensins [14], plant defensins are able to inhibit bacteria and fungi [10]. The high antifungal activity of plant defensins underscores the significance of fungal pathogens in the plant world, which differs from the high antibacterial activity of animal defensins [14]. Defensins have been identified in locations of first contact and entry by plant pathogens, including peripheral cell layers, xylem, and stomatal cells and in cells lining the substomatal cavity. They have been isolated from tubers, leaves, pods, seeds, and flowers [10]. Defensin gene expression can be developmentally regulated or influenced by external stimuli. Pea, tobacco, radish, and* Arabidopsis* have defensin genes that are expressed upon pathogen infection.

There are four defensin groups, which are classified by structural properties. Group I inhibits Gram-positive bacteria and fungi, group II inhibits fungi, group III inhibits Gram-positive bacteria and Gram-negative bacteria, and group IV inhibits Gram-positive bacteria, Gram-negative bacteria, and fungi. Importantly, there has been no reported toxicity of plant defensins to animal or plant cells [10] which is very significant from a food safety standpoint should these antimicrobials be leveraged as biopreservatives. The mode of action of antifungal defensins is potentially dependent on electrostatic interactions between hyphal membranes and peptides that cause a rapid Ca^2+^ influx and K^+^ efflux [17].

IbAMP1 from the seeds of* Impatiens balsamina* represents one of the smallest pAMPs with only 20 residues (Table 1) and two disulfide bonds. IbAMP1 has been shown to be active against fungi, Gram-positive bacteria, and Gram-negative bacteria at micromolar levels [18–21]. Wu et al. [22] have demonstrated the concentration-dependent effect of Ib-AMP1 on the cell membrane of Shiga toxin-producing* E. coli* O157:H7. They showed that Ib-AMP1 exerted its bactericidal activity by interfering with outer and inner membrane integrity permitting efflux of ATP and interfering with intracellular biosynthesis of DNA, RNA, and protein [22].

López-Solanilla et al. [16] used an* in vitro* method that showed that potato defensin was only weakly inhibitory to* L. monocytogenes* and* L. ivanovii* at 37°C (MICs are listed in Table 2). However, at 20°C, the two species were resistant. Potentially, this shows an adaptive technique by* L. monocytogenes* and* L. ivanovii* to improve survival when in their primary natural habitat of decaying plant matter-filled soil, which is about 20°C.

### 3.3. Lipid Transfer Proteins (LTPs)

These peptides were once thought to be involved in the transfer of lipids between organelles but have been shown to be involved in plant defense. They seem to have an important role in pathogen defense as well as during low temperature and salt stress. LTPs and defensins can synergistically inhibit fungal and bacterial growth in plants [17]. They are expressed in many areas of a plant, especially in exposed surfaces and in vascular tissues. LTPs have been isolated from barley (Table 1) [10], maize, spinach,* Arabidopsis*, broccoli, and radish and have demonstrated some specificity [10].

There is limited research on the mode of action of plant LTPs.* In vitro* research suggests plant LTPs function in plant defense against pathogens based on their ability to inhibit microbial growth. However, there is little direct evidence of the basis of their antimicrobial activity and, unlike other AMPs, they are thought to have many other roles* in vivo*. Ha-AP10, a LTP, completely inhibits the germination of spores at a concentration of 40 *μ*g/mL. Regente et al. [23] demonstrated that Ha-AP10 acts as a fungicidal compound by not only inducing liposome leakage but also modifying the permeability in* Fusarium solani* spores. Although other factors may also contribute to the fungicidal activity, the membrane permeabilization mechanism is common to other pAMPs. In addition, the authors demonstrated that Ha-AP10 was able to permeabilize fungal cells in media containing 1 mM CaCl_2_. This is significant because it more closely represents environmental conditions since the physiological concentration of free Ca^2+^ is 0.1–1 mM in the apoplast, where the fungal-plant contact is likely to occur. Furthermore, the authors demonstrated the selective toxicity of Ha-AP10 for fungal cells over plant (potato host cells). By conducting a follow-up experiment that used model membranes with encapsulated fluorescent probes, the authors hypothesized that this differentiation was due to the composition of phospholipids in the plant and fungal membranes. The activity of Ha-AP10, a cationic and hydrophobic peptide, may therefore be mediated by its electrostatic interaction with anionic membrane phospholipids [23].

### 3.4. Myrosinase-Binding Proteins (MBPs)

Myrosinase (EC 3.2.3.1) is a glucosinolate-degrading enzyme mainly found in the Brassicaceae special idioblasts, myrosin cells [24]. MBPs are involved in plant development and defense activities, primarily against pathogens and insects. The amino acid features of the MBP1 from Arabidopsis are shown in Table 1 [17, 25]. It has been discovered that the potential MBP mode of action is to act as ionophores over microbial membranes [17]. The mechanism of action has been proposed by Capella et al. [25] to be the hydrolysis of glucosinolates by myrosinase enzymes producing molecules with diverse modes of action against fungi, bacteria, and insects.

### 3.5. Hevein- and Knottin-Like Peptides

Both of these peptides inhibit fungi and Gram-positive bacteria* in vitro*. They have been primarily isolated from seeds, but hevein-like peptides (HTPs) have been found in other tissues as well [10]. Hevein is a small chitin-binding peptide that was initially isolated from rubber latex (Table 1) [26]. This peptide can be used as a fungicide but it may be allergenic, presenting a significant barrier from a safety and labeling standpoint should this peptide be considered as a food preservative. The mechanism of action of HTPs is hyphal penetration that leads to cell burst [17].

### 3.6. Snakins

Snakins are antimicrobial peptides that have been isolated from potatoes (Table 1) [27]. The snakin-1 peptide is active against fungi and Gram-positive and Gram-negative bacteria at concentrations less than 10 *μ*M [10]. The peptide is able to aggregate bacteria* in vitro* but does not mediate leakage or aggregation of artificial liposomes at low- or high-salt concentrations and does not destroy lipid membranes [17]. The mechanism of action of this peptide has yet to be elucidated.

Using an* in vitro* method, López-Solanilla et al. [16] showed that a snakin peptide had a strong inhibitory effect on* L. monocytogenes* and* L. ivanovii* (MICs are listed in Table 2). The* Listeria* species exhibited differential susceptibility to various pAMPs, which could be potentially linked to the differential fate of* Listeria* in different areas of the plant.

### 3.7. Cyclotides

Plant cyclotides do not have N- or C-termini because this peptide has a cyclic structure, which serves an important role in the peptide's activity and stability. The structure is a head-to-tail backbone with six conserved cysteine residues, forming a knot motif. They can be classified into two subfamilies: Mobius and bracelet. Mobius cyclotides have a twist formation in the backbone whereas bracelet cyclotides do not have a twist. A third group in the cyclotide family consists of proteinase inhibitors that were isolated from* Momordica cochinchinensis, *and another cyclotide structure, kalata B8, isolated from* Oldenlandia affinis,* seems to be a hybrid of the Mobius and bracelet subfamilies [28]. Kalata B1 is listed in Table 1 [28].

These peptides serve various plant defensive roles, including cytotoxicity to plant tumor cells, antiviral and insecticidal activities, and proteinase inhibition. Some also exhibit antibacterial activity, with peptide kalata B1 and circulin A active against Gram-positive bacteria, such as* Staphylococcus aureus*, and circulin B and cyclopsychotride active against both Gram-positive and Gram-negative bacteria [28]. Pränting et al. [29] conducted research to determine the antibacterial activity of various cyclotides against several Gram-positive and Gram-negative bacterial strains (MIC values are listed in Table 1). From the five evaluated cyclotides, cycloviolacin O2 (cyO2) was determined to be the most potent antibacterial cyclotide, showing high efficacy against Gram-negative bacterial species, including* E. coli*. This activity is significant given the evidence that other antimicrobial agents, such as nisin, are generally less active against Gram-negative species. However, the mode(s) of action for cyclotides have not yet been elucidated. More research is needed to determine mechanism(s) of action and further biological functions of cyclotides, but these compounds have great potential to serve as novel antibiotics and antiviral therapies to control infectious diseases [28].

### 3.8. Peptides from Hydrolysates

Plant protein hydrolysates can be a source of bioactive peptides [13]. Hydrolysis is either done enzymatically or by acids. Hydrolysates from leguminous plants are particularly favored as they are parts of the food ingredients for many countries in the world [13]. As summarized by Salas et al. [13], the enzymatic hydrolysates from common bean varieties of* Phaseolus* demonstrated antimicrobial activities against* S. aureus* and* Shigella flexneri* with MIC values in the range of 0.1 to 0.99 mg/mL. Another report has shown that the alcalase hydrolysates of rapeseed (*Brassica napus*) protein inhibited the protease activity of human immunodeficiency virus (HIV) that was expressed in* E. coli* cells [30].

Some industry by-products represent another source of bioactive peptides that possess antimicrobial properties. For example, the palm kernel expeller (PKE) is produced after palm kernel oil production [31]. Tan et al. tested the efficacy of the purified alcalase- and tryptic-hydrolysates of PKE, PAH, and PTH on* Bacillus cereus* [31, 32]. It was shown that both PAH and PTH disrupted the membrane integrity of* B. cereus*, allowing efflux of K^+^, depleted the ATP molecules, and inhibited the intracellular macromolecule metabolism especially the RNA of the bacterium.

## 4. Structure of pAMPs Related to Their Modes of Action

Plant AMPs have similar physical properties but diverse primary amino acid sequences. In addition, pAMPs have a range of secondary structures, but there are at least four major themes: loop structures, amphiphilic peptides with two to four *β*-strands, amphipathic *α*-helices, and extended structures [11]. However, there are peptides that do not fit into this structure classification, such as many bacterially produced peptides that have two domains, one of which is *α*-helical and the other of which has a *β* structure. In addition, there is little scientific literature describing the tertiary structures of pAMPs. However,* in silico* analyses have shown that pAMPs have similarities in their three-dimensional structures [12].

The antibacterial mode of action for most pAMPs involves cell membranes of targeted organisms and is driven by net positive charge, flexibility, and hydrophobicity to enable interaction with bacterial membranes [11]. Although it was originally thought that the sole mode of action was permeabilization of the bacterial cell membrane, research suggests there may be alternative modes of action or that the pAMPs act upon multiple cell targets. However, interaction with the bacterial cell membrane is critical. There are several models in the literature that illustrate interaction at the cell membrane, each of which uses a different intermediate that leads to either formation of a transient channel, translocation across the membrane, or micellization or dissolution of the membrane. The modes of action are therefore either membrane acting (permeabilizing) or nonmembrane acting (nonpermeabilizing) since translocation does not cause membrane disruption but allows entrance to target essential intracellular processes. In addition, a peptide may target both the cell membrane and intracellular components [11].

The antifungal mode of action was first thought to only involve cell lysis or interference with the synthesis of the fungal cell wall. However, research indicates additional modes of action, including permeabilization, binding to ergosterol/cholesterol in the membrane, depolymerization of the actin cytoskeleton, and targeting intracellular organelles, such as mitochondria [11]. The mode of action of some antifungal peptides is still debated amongst researchers. Plant antimicrobial peptides with predominantly antifungal efficacy tend to be rich in polar and neutral amino acids. The mode of action by plant defensins against fungi has recently been reviewed by Vriens et al. [33].

Antiviral activity is often related to a direct effect on the viral envelope or related to the viral adsorption and entry process [11]. Some antiprotozoal modes of action are similar to antibacterial, antifungal, and antiviral mode of action, such as cell membrane disruption via pore formation or direct interaction with the lipid bilayer. However, there are conflicting reports found in the literature which indicate that antiprotozoal activity may be dependent on peptides that are different from viral, fungal, and bacterial activities [11].

## 5. Resistance to pAMPs

Microbial resistance to antimicrobial agents used in food preservation and sanitation is a major concern. Antibiotic resistance is generally caused by transfer of resistance genes between bacterial cells [34]. Laboratory and clinical studies have determined that resistance to AMPs is less likely than resistance to conventional antibiotics [35]. This is likely due to their membrane-targeting mechanism of action that is more difficult to develop resistance to antibiotics, which generally target macromolecular synthesis (DNA, RNA, and protein) [36]. However, it has been demonstrated that specific genes can confer resistance to pAMPs. For example, the* pagP* gene increases resistance to the bactericidal effects of some antimicrobial peptides in* Salmonella* [37]. Changes to the targeted organism's cell membrane may also lead to resistance. It has been difficult to develop resistant strains from previously sensitive strains to particular antimicrobial peptides [6]. This is an ongoing area of research.

It is imperative to note that commensal bacteria, which are beneficial to the host organism, such as* Lactobacillus* in the intestines of humans, are relatively resistant to the action of endogenous antimicrobial peptides [35]. This type of resistance and AMP specificity suggests that pAMPs could potentially be used in food application after further toxicology studies are conducted.

## 6. Phytochemicals

Phytochemicals are nonnutritive plant components that confer organoleptic properties and serve as antimicrobial agents. The concentration, composition, structure, and functional groups serve an important role in determining antimicrobial activity. Phenolic compounds are generally the most effective [7]. Based on their chemical structures, they may be divided into different categories including simple phenolic compounds, flavonoids, quinones, tannins, and coumarins. The most important phytochemicals used as food preservatives are essential oils, which have been used by humans across the continents since ancient times. Some alkaloids from plants have also been used as antimicrobials in food. Recently, many different phytochemicals have been listed by Negi [38] and their antibacterial activities have been summarized. The antifungal and antifungal toxin activities from various plant extracts including phenolic compounds and essential oils have also been recently reviewed [39]. Polyphenolic compounds from fruits such as cranberry, pomegranate, blueberry, raspberry, and grape were also summarized in 2014 for their antiviral activities against human enteric viruses [40]. Here we briefly review these phytochemicals for their antimicrobial activities in food applications with some examples.

### 6.1. Simple Phenolic Compounds

Simple bioactive phytochemicals are comprised of a single substituted phenolic ring [41]. There is some evidence that the sites and number of hydroxyl groups on the phenolic ring are related to the degree of toxicity to microorganisms, with increased hydroxylation resulting in increased toxicity. It has also been suggested that higher oxidation confers greater inhibition [1]. The mode of action is enzyme inhibition by the oxidized compounds. Phenolic compounds are known to alter microbial cellular permeability, resulting in loss of macromolecules, and interact with membrane proteins, causing structural changes [39]. A simple phenol example is caffeic acid, which is found in thyme and tarragon and is active against fungi, viruses, and bacteria. Eugenol is a phenolic compound found in clove oil that is active against bacteria and fungi [1].

### 6.2. Flavones, Flavonols, and Flavonoids

Flavones are phenolic compounds with one carbonyl. Flavonols are phenolic compounds with a carbonyl and a 3-hydroxyl group. Flavonoids are hydroxylated phenolic structures with a C3–C6 aromatic ring linkage. They are effective against many microorganisms because of their ability to bind to and inactivate proteins and to complex with bacterial cell walls. Catechins provide the antimicrobial activity in oolong teas. The green tea polyphenol, epigallocatechin-3-gallate, was shown to be antiviral against hepatitis B virus replication* in vitro* [42]. Unlike simple phenolic compounds, the degree of hydroxylation does not predict the level of toxicity to microorganisms [1].

### 6.3. Quinones

Quinones are aromatic rings with two carbonyls, providing a stable source of free radicals. In addition to serving as antioxidants, they are potent antimicrobial compounds. Similar to flavones, flavonols, and flavonoids, the antimicrobial mode of action is to bind to and inactivate proteins. In addition, they may make substrates unavailable to the microorganism [1]. The 6-(4, 7-dihydroxy-heptyl) quinone isolated from the leaves of* Pergularia daemia* (Forsk.), a traditional medicinal plant, was shown to be effective against several food-contaminating pathogens including* Bacillus subtilis, Staphylococcus aureus*, and* Escherichia coli* [43].

### 6.4. Tannins and Coumarins

Tannins are polymeric phenolic substances that are divided into hydrolysable and condensed tannins (also known as proanthocyanidins). The latter are based on flavonoid monomers and hydrolysable tannins are based on gallic acid. Tannins may be formed by polymerization of quinones or by condensation of flavan derivatives. Their antimicrobial mode of action is similar to that of quinones and they have been shown to be toxic to bacteria, yeasts, and some fungi [1]. Tannins naturally occur in many fruits, nuts, and seeds. A recent review by Lipińska et al. [44] shows that the hydrolysable ellagitannins found in pomegranate, strawberry, blackberry, raspberry, walnuts, almonds, and seeds exhibit antimicrobial activity against fungi, viruses, and, importantly, bacteria, including antibiotic-resistant strains such as methicillin-resistant* Staphylococcus aureus* (MRSA). Additionally, a recent article has comprehensively reviewed the antimicrobial activities of bioactive components from berries including flavonoids (anthocyanins, flavonols, and catechins), phenolic acids, stilbenes, and tannins [45].

Coumarins are phenolic structures comprised of a fused benzene and alpha-pyrone ring. Although toxic to some animals, they have been shown to have species-dependent metabolism, with toxic coumarin derivatives excreted in human urine without adverse health effects. A recent review summarizes not only the anti-inflammatory, anticoagulant, anticancer, antihypertensive, antitubercular, anticonvulsant, antiadipogenic, antihyperglycemic, antioxidant, and neuroprotective properties of coumarins but also their antibacterial, antifungal, and antiviral activities [46].

### 6.5. Essential Oils

Essential oils, or terpenes, are secondary metabolites and are based on an isoprene structure. They are volatile compounds that provide the fragrance of plants and are mainly responsible for the flavor and aroma of spices. Terpenoids contain additional elements, such as oxygen, and confer activity against bacteria, fungi, protozoa, and viruses [1]. Research has shown that essential oils have anti-inflammatory, bactericidal, antiviral, and anticancer effects and possess antioxidant activity [47]. For example, Delaquis et al. [48] determined that the essential oil of cilantro was particularly effective against* Listeria monocytogenes,* potentially because of long chain alcohols and aldehydes since the antimicrobial properties of alcohols are known to increase with molecular weight. A review by Seow et al. [49] has included 47 different essential oils as antimicrobials. The antibacterial and antifungal activities of these essential oils have been listed. As essential oils contain highly diverse groups of phytochemicals, their antimicrobial modes of action have been suggested to involve multiple targets. The unique hydrophobicity features of essential oils render their abilities to react with lipids on the bacterial cell membranes, increasing the membrane permeability and disturbing the original cell structure [50, 51]. The clover essential oil has been shown to cause an extensive lesion of fungal cell membrane [52]. Essential oil has also been shown to inhibit viral protein synthesis at multiple stages of viral infection and replication [53].

### 6.6. Alkaloids

Alkaloids are heterocyclic nitrogen compounds and have demonstrated limited microbicidal activity as well as possessing an antidiarrheal activity. An example of an alkaloid is berberine [1]. Berberine is the main antibacterial substance of rhizoma Coptidis (*Coptis chinensis* Franch) and cortex Phellodendri (*Phellodendron amurense* Ruprecht) [54]. The MICs of berberine against methicillin-resistant* Staphylococcus aureus* (MRSA) bacteria ranged from 32 to 128 *μ*g/mL. Ninety percent inhibition of MRSA was obtained with 64 *μ*g/mL or less of berberine.

## 7. Stability of Plant Antimicrobial Compounds

The effect of food processing on plant-derived antimicrobial compounds must be evaluated. Plant-derived foods are often exposed to acidic or alkali conditions or high heat during processing to destroy microorganisms to enhance microbial food safety. The aforementioned conditions are used to peel fruits and vegetables and to recover proteins from cereals and legumes [55]. However, these conditions may destroy natural plant antimicrobial compounds, which serve as defense mechanisms to the plant and have potential to serve as natural antimicrobial compounds against human pathogens.

Research by Friedman and Jürgens [55] indicated that the chemical structure of phenolic compounds has a significant effect on their susceptibility to destruction at alkali conditions. By using ultraviolet spectroscopy, they determined that gallic acid, caffeic acid, and chlorogenic acid were not stable in high pH and that the spectral transformations were not reversible when the pH was reduced back to neutral conditions. The phenolic OH groups were hypothesized to be primarily responsible for the spectral changes since ferulic acid (with one OH group) was more stable in high pH versus caffeic acid (with two OH groups) and gallic acid (with three OH groups). Furthermore, the ionized and resonance forms of multiring structures conferred more resistance to high pH versus monoring structures. Therefore, multiring catechin, epigallocatechin, and rutin had less spectral transformations at high pH conditions versus monoring gallic acid, caffeic acid, and chlorogenic acid. This research provides a foundation that suggests structural elements that may confer stability at alkali conditions. It is critical to determine whether natural antimicrobial compounds are stable in food processing conditions in order to determine their feasibility as alternative antimicrobial compounds to foodborne pathogens in food systems.

In addition to this foundational research under artificial conditions, it is critical for natural antimicrobials to be tested in food systems. For example, solubility and food constituents, such as proteins and lipids, could potentially impact efficacy and stability of plant-derived antimicrobial compounds. Research conducted by Aureli et al. [56] indicated that thyme essential oil reduced viable* Listeria monocytogenes* L28 cells in a meat matrix but noted that there was a decreased efficacy in the food system compared to* in vitro* testing. Pränting et al. [29] reported that the antibacterial efficacy of the pAMP cyclotide cyO2 was reduced in media containing salt, which suggests that this peptide would be less efficacious as a preservative in a high-salt food. The authors do note, however, that even though some research has indicated similar influence of media composition on antibacterial efficacy of AMPs, the AMPs were still active against bacteria in biological systems. It is essential that the antimicrobial compounds effectively reduce pathogenic bacteria to allowable limits or completely inactivate pathogens.

Because pasteurization can affect organoleptic and nutritional properties and increase processing costs and postpasteurization contamination can occur, Friedman and Jürgens [55] conducted a study to assess the stability of a naturally occurring antimicrobial, chlorogenic acid, in apple juice. The authors determined that the phenolic compound, chlorogenic acid, was stable in the low pH, heat treatment, and storage of apple juice, offering a promising candidate to combat contamination by* E. coli* O157:H7 and* Salmonella* Typhimurium of nonfermented apple juice products. Payne et al. [57] determined that the phenolic compounds propyl paraben (propyl ester of p-hydroxybenzoic acid) and tertiary butylhydroquinone (TBHQ) were significantly more effective than potassium sorbate, a commonly used antimicrobial, against* Listeria monocytogenes* in a model milk system containing 10% nonfat milk solids at 35°C. However, TBHQ was inconsistent in its activity. Although the authors suggest that this study indicates that inhibition would be achieved at refrigeration temperatures, it seems that there may have been an error in conversion of Celsius to Fahrenheit, since 35°C is much greater than refrigeration temperatures. The authors do raise a valid point because it is important to conduct studies at the proper storage temperature of the food product in order to mimic normal food shelf conditions and because temperature greatly affects the survival rate of foodborne pathogens.

## 8. Applications of Plant Antimicrobial Compounds in Food Industry

For the food industry, plant antimicrobial compounds have potential use as biopreservatives and bioinsecticides, with potential use for development of genetically modified crop plants with increased disease resistance as well as use against foodborne pathogens. In fact, research has already shown the effectiveness of plant compounds against virulent foodborne pathogens. For example, essential oils have been used to help control* Listeria monocytogenes.* Aureli et al. [56] used a paper disc diffusion method to demonstrate that 12 out of 32 essential oils were active against* Listeria monocytogenes*, with clove, cinnamon, pimento, origanum, and thyme showing the greatest inhibition. However, the application of plant antimicrobial compounds for controlling growth of foodborne pathogens must incorporate the range of activity against the microorganisms of concern associated with a particular product. For example, essential oils are typically more effective against Gram-positive than Gram-negative bacteria. But some, such as clove and cinnamon, have been shown to be effective against both [3]. Olasupo et al. [58] demonstrated that 5 natural organic compounds were effective against the Gram-negative bacteria* E. coli* and* Salmonella* Typhimurium with efficacy in the following order: thymol > carvacrol > eugenol > cinnamic acid > diacetyl. Table 1 shows the MIC values of various antimicrobial agents against pathogenic bacterial targets at various conditions. This table illustrates that the efficacy of natural antimicrobials is impacted by environmental conditions and antimicrobial agents have a wide range of activity against bacterial targets. This information must be fully verified for a particular food product when considering the use of plant-derived antimicrobials in the food product. Although this review focused on efficacy against pathogenic organisms, it should be noted that the common spoilage bacteria,* Pseudomonas*, are generally resistant to plant antimicrobials due to the production of exopolysaccharide layers that offer protection and delay penetration of antimicrobials [3].

Another potential use of antimicrobial peptides in the food industry is replacement of antibiotics used in animal production to increase feed efficiency. Although there are some conflicting reports in the literature, Jin et al. [59] determined that pigs fed increasing levels (0 to 600 ppm) of refined potato protein (RPP) from* Solanum tuberosum* L. cv. Gogu Valley demonstrated linear improvements in performance and a linear decrease in fecal and intestinal bacteria, suggesting that RPP was effective at higher levels. Since the potato tubers of Gogu Valley are known to contain the AMP potamin-1 (PT-1), the authors suggest that the AMP may have caused the decrease in microbe numbers, contributing towards increased performance. Given the linear effects demonstrated with increasing levels of RPP in the pigs' diets, future studies that evaluate higher levels of RPP in pigs (as well as other production animals) should be completed to more fully elucidate the potential of RPP as an alternative to antibiotics. Furthermore, other AMPs should be evaluated as potential performance enhancers and chemical modification or encapsulation methods should be evaluated to prevent degradation by proteolytic enzymes within the digestive system of production animals [6]. However, it is known that cyclotides are stable against proteases, such as pepsin and trypsin, and their cyclic structure confers protection against exopeptidases [29].

## 9. Technologies by Which Plant Antimicrobials Can Be Delivered

There are various methods by which plant antimicrobials could be delivered. The most suitable methods must incorporate cost-effectiveness, stability, and efficacy of the compound under processing, transportation, and storage conditions. A recent technology incorporates antimicrobials into packaging materials instead of the food itself. This technique offers the advantage of concentrating the antimicrobial at the surface of the food product, which is where potential pathogens grow, and reduces obstruction from food particles [7].

Microencapsulated antimicrobial agents incorporated in food packaging have been demonstrated to successfully destroy a range of microorganisms, offering a controlled-release preservation technique. This application is a type of active packaging (AP), in which the conditions of the packaged food are changed to better preserve the sensory attributes, safety, and shelf-life of the product. Since microbial contact primarily occurs on the surface of a packaged food, antimicrobial activity should be focused on solid or semisolid surfaces by either indirect contact using antimicrobial volatiles or via direct contact between the antimicrobial package and the food surface. Research has successfully incorporated antimicrobial peptides, such as bacteriocins, as well as phytochemicals, such as essential oils, into packaging materials [60].

Guarda et al. [60] demonstrated the antimicrobial activity of microencapsulated carvacrol and its isomer thymol, which are phenolic compounds that are major components of essential oils with known antimicrobial activity. By creating emulsions with the compounds and applying them to a polymer film, the authors demonstrated their antimicrobial activity by the agar plate method. The minimal inhibitory concentration (MIC) of thymol was 125–250 ppm and the MIC of carvacrol was 75–375 ppm against both pathogenic and nonpathogenic microorganisms:* Escherichia coli* O157:H7,* Staphylococcus aureus*,* Listeria innocua*,* Saccharomyces cerevisiae*, and* Aspergillus niger*. The highest synergism was determined to be 50% of each compound and various concentrations were studied to determine the required concentration for the most resistant microorganism,* E. coli*, based on zones of inhibition. The authors found similar findings to others in the literature that Gram-positive bacteria were more sensitive than Gram-negative bacteria, such as* E. coli*, potentially due to the outer membrane that surrounds the lipopolysaccharide wall inhibiting the penetration of the hydrophobic phenolic compounds. The required concentration of the antimicrobial agent to confer antimicrobial activity therefore depends on the type of targeted organism in a given food product. Furthermore, it is important to note that loss of antimicrobial activity may occur during preparation of the packaging. Lower losses were observed in the coating process utilized [60] than high-temperature processes, such as extrusion blow-molding. The authors noted that the release rate of the microcapsules was lower and more controlled as compared to films with antimicrobial agents directly incorporated into the matrix, potentially due to the affinity of the carrier, gum arabic, to a polar compound and the good film forming capability of gum arabic. This research provides a strong foundation for the use of these GRAS approved materials in active packaging, but more research is required on the use of this packaging on food systems.

Chitosan is a hydrophilic polymer that is obtained commercially by N-deacetylation of chitin. Chitosan exhibits some antimicrobial activity against fungi, algae, and some bacteria. Antimicrobial efficacy is influenced by the type of chitosan, molecular weight, and environmental conditions. Chitosan is limited by its insolubility in water, high viscosity, tendency to coagulate with proteins, and poor solubility at high pH. However, water-soluble salts can be formed by neutralization with acids. The exact antimicrobial action of chitosan is debated based on review of the literature, but it has been suggested that chitosan interacts with negatively charged microbial cell membranes, leading to cellular leakage. The polymer also acts as a chelating agent that binds trace metals, inhibiting toxin production and microbial growth. Research is also being conducted to determine its ability to elicit natural plant defenses when applied to plant tissues or cultured plant cells [61].

Chitosan is a biopolymer that is safe for human consumption and has several effective delivery methods, including the use as a seed treatment and as an edible antifungal coating material for postharvest produce. Chitosan films are semipermeable, durable, long-lasting, natural, inexpensive, and nontoxic and have been successfully used to delay decay of various fruits and vegetables potentially due to decreased rates of respiration, delay of ripening from the reduction of ethylene and carbon dioxide production, and fungal inhibition. Additional antimicrobial agents can be applied to chitosan films so that they serve as an active type of packaging, releasing the biopreservatives in a controlled manner onto the food surface to inhibit microbial growth. N,O-Carboxymethyl chitin films have been approved for use in fruits in both USA and Canada. Further research is needed to determine novel derivatives of chitosan with increased antimicrobial activity [61].

Plant antimicrobials can be delivered via plant extracts or consumed whole. The literature generally cites that spice extracts are less antimicrobial than the whole spice. For example, Lachowicz et al. [62] used an agar well diffusion method to demonstrate that essential oils extracted from five different varieties of* Ocimum basilicum* L. plants showed equivalent or better antimicrobial activity against a range of Gram-positive and Gram-negative bacteria, yeasts, and molds compared to the purified components linalool and methyl chavicol either separately or together. In contrast, Delaquis et al. [48] determined that fractions of dill and cilantro oil had greater antimicrobial activity than the whole oil. The latter authors aptly suggest that essential oils, which are naturally variable in composition, could more reliably be used as preservative agents if the concentrations of antimicrobial components could be adjusted to consistent levels for the needed spectrum and strength of microbial inhibition. More research is required in this area.

## 10. Regulation of Plant-Derived Antimicrobials for Food Application

The exact number of plant-derived antimicrobials approved for food application globally is difficult to discern due to the limited amount of information available. Food additives are closely controlled by legislation worldwide, but there is little agreement between countries regarding food additives that are safe, permitted concentrations, and specific permitted food uses [63]. In the US, the Food and Drug Administration (FDA) evaluates the safety of unapproved food additives to determine whether they should be approved. The evaluation includes the amount of the substance that would normally be consumed as well as short- and long-term health effects and other safety considerations. When a food additive is approved, the FDA issues a regulation that may include the types of foods in which it can be used, the maximum amount allowed, and its proper identification on food labels. These regulations are published in the Title 21 of the* Code of Federal Regulations*. According to the FDA's Guidance for Industry: Antimicrobial Food Additives, the FDA has regulatory authority over food additives. However, the Environmental Protection Agency (EPA) sets tolerances for pesticide chemicals and pesticide chemical residues in or on foods, which are enforced by the FDA. It should be noted that antimicrobials applied to, or included in, food packaging materials are excluded from the definition of “*pesticide chemical*” and thus are regulated as food additives by FDA.

Although regulation varies worldwide, other countries have similar departments or agencies in place to evaluate the safety and provide guidance and regulation on food additives. In Europe, there are relatively few compounds that are allowed as food preservatives, and these are primarily organic acids [63]. There is a strict protocol in place in order for food additives to be approved for human consumption. When applying for authorization of a new food additive, an applicant submits a formal request to the European Commission, which is the executive body of the European Union, and includes information on the substance, including scientific data concerning safety. Upon acceptance of the application, the Commission requests the European Food Safety Authority (EFSA) to issue an opinion on the safety of the substance for its intended uses. In addition to carrying out safety evaluations of new food additives before they can be authorized for use in the European Union (EU), EFSA reviews certain food additives that have new scientific information and/or changing conditions (http://www.efsa.europa.eu/en/topics/topic/additives.htm). Similarly, the Chinese government employs the China Food Additives Association (CFAA) as the only registered nationwide food additive and ingredients industry organization to make evaluations and assist the government in making regulations in the food and food additives industry (http://www.cfaa.cn/english.htm).

Given the strict regulation and the relatively limited* in vivo* research on pAMPs, a literature review and a search of the web pages of the governmental agencies listed above yielded no known approved plant AMPs in those countries for food application. The agencies are constantly evaluating new potential food additives, including biopreservatives, so regulation of plant-derived antimicrobials can be expected to be seen in the future.

## 11. Conclusion

Food processing and some preservation techniques, such as heating, may alter food's nutritional or organoleptic properties. Microbial resistance to current antimicrobial compounds has increased in recent years worldwide; therefore, alternative compounds must be investigated and developed. This review has cited many of the benefits and lists some of the current hurdles of implementing plant-derived antimicrobials in food application. Most significantly, despite extensive* in vitro* research of plant-derived antimicrobials, there are limited* in vivo* studies, yielding knowledge about the toxicology of the extensive repertoire of compounds. Future toxicology research will aid the governmental food safety agencies in their evaluation and regulation of these compounds for food application.

Because of variation in stability and efficacy to various food processing parameters and food systems, it is critical that plant-derived antimicrobials be selected and delivered so that they are active against potential pathogens in particular food and are stable throughout the food's shelf life. Effects of these compounds in combination with other compounds or techniques must be more thoroughly investigated. For example, hevein- and knottin-like peptides are active against Gram-positive bacteria and fungi. However, research should be conducted to verify if they would also be active against Gram-negative bacteria if chelators that perturb the outer membrane of Gram-negative bacteria were also present. In addition to these scientific evaluations, sensory studies must be conducted to ensure that the organoleptic properties of a food are not impacted by the natural antimicrobial peptides.

Given the consumer demand for more natural products and the growing need for alternative preservatives to ensure food safety, it is imperative that plant-derived antimicrobial compounds be fully assessed for their feasibility for food application. This new field of research has great potential for more evaluation to meet regulatory requirements and to fully elucidate the possibility of employing antimicrobials from the extensive source of plants worldwide.

## Figures and Tables

**Table 1:** Molecular structures of selected pAMPs.

pAMP	Amino acid (AA) sequence	Plant source	Reference
Thionin-*α*1	KSCCRSTLGRNCYNLCRARGAQ KLCAGVCRCKISSGLSCPKGFPK	*Triticum aestivum* (wheat)	[15]

IbAMP1	QWGRRCCGWGPGRRYCVRWC	*Impatiens balsamina *	[22]

Lipid transfer protein 2	AITCGQVSSALGPCAAYAKG SGTSPSAGCCSGVKRLAGLA RSTADKQATCRCLKSVAGA YNAGRAAGIPSRCGVSVPY TISASVDCSKIH	*Hordeum vulgare* (barley)	[10]

MBP1	153 AA repeats with 13 AA motif: SGKGTDSGSST(K/Q)D 8 AA motif: GSQGGQGG	*Arabidopsis* *thaliana *	[17, 25]

Hevein	EQCGRQAGGKLCPNNLCCSQWG WCGSTDEYCSPDHNCQSNCKD	*Hevea* *brasiliensis *	[26]

Snakin1	GSNFCDSKCKLRCSKAGLADR CLKYCGVCCEECKCVPSGTYG NKHECPCYRDKKNSKGKSKCP	*Solanum* *tuberosum* (potato)	[27]

Kalata B1	CGETCVGGTCNTPGCTCSWPV CTRNGLPV	*Oldenlandia* *affinis *	[28]

**Table 2:** MIC values of antimicrobial agents.

AMP agent	Bacterial target	MIC	Recorded condition	Reference
Potato defensin	*L. monocytogenes *	>25 *μ*g/mL	24 h at 37°C	[14]
Thionins	*L. monocytogenes *	2 *μ*g/mL	24 h at 37°C	[14]
Snakin	*L. monocytogenes *	10 *μ*g/mL	24 h at 37°C	[14]
Lipid transfer protein	*L. monocytogenes *	no effect	24 h at 37°C	[14]
Potato defensin	*L. ivanovii *	>25 *μ*g/mL	24 h at 37°C	[14]
Thionins	*L. ivanovii *	5 *μ*g/mL	24 h at 37°C	[14]
Snakin	*L. ivanovii *	10 *μ*g/mL	24 h at 37°C	[14]
Lipid transfer protein	*L. ivanovii *	>25 *μ*g/mL	24 h at 37°C	[14]
Carvacrol	*Salm.* Typhimurium	1 m/mol	16 h at 37°C	[34]
Cinnamic acid	*Salm.* Typhimurium	7.5 m/mol	16 h at 37°C	[34]
Diacetyl	*Salm.* Typhimurium	12.5 m/mol	16 h at 37°C	[34]
Eugenol	*Salm.* Typhimurium	3.0 m/mol	16 h at 37°C	[34]
Thymol	*Salm.* Typhimurium	1.0 m/mol	16 h at 37°C	[34]
Carvacrol	*E. coli *	1.5 m/mol	16 h at 37°C	[34]
Cinnamic acid	*E. coli *	5.0 m/mol	16 h at 37°C	[34]
Diacetyl	*E. coli *	7.5 m/mol	16 h at 37°C	[34]
Eugenol	*E. coli *	2.5 m/mol	16 h at 37°C	[34]
Thymol	*E. coli *	1.2 m/mol	16 h at 37°C	[34]
Thymol	*E. coli *	250 ppm	48 h at 37°C	[35]
Carvacrol	*E. coli *	375 ppm	48 h at 37°C	[35]
Cyclotide CyO2	*E. coli *	2.2 *μ*M	37°C	[20]
Cyclotide Kalata B1	*E. coli *	≥100 *μ*M	37°C	[20]
Cyclotide Kalata B2	*E. coli *	≥35 *μ*M	37°C	[20]
Cyclotide Vaby A	*E. coli *	32.5 *μ*M	37°C	[20]
Cyclotide Vaby D	*E. coli *	50 *μ*M	37°C	[20]
Cyclotide CyO2	*S. aureus *	>50 *μ*M	37°C	[20]
Cyclotide Kalata B1	*S. aureus *	>100 *μ*M	37°C	[20]
Cyclotide Kalata B2	*S. aureus *	35 *μ*M	37°C	[20]
Cyclotide Vaby A	*S. aureus *	>90 *μ*M	37°C	[20]
Cyclotide Vaby D	*S. aureus *	>90 *μ*M	37°C	[20]
Thymol	*S. aureus *	250 ppm	48 h at 37°C	[35]
Carvacrol	*S. aureus *	225 ppm	48 h at 37°C	[35]
Cyclotide CyO2	*S. enterica *	8.75 *μ*M	37°C	[20]
Cyclotide Kalata B1	*S. enterica *	>100 *μ*M	37°C	[20]
Cyclotide Kalata B2	*S. enterica *	>35 *μ*M	37°C	[20]
Cyclotide Vaby A	*S. enterica *	90 *μ*M	37°C	[20]
Cyclotide Vaby D	*S. enterica *	>90 *μ*M	37°C	[20]
